# Multi-omics analysis identified macrophages as key contributors to sex-related differences in ulcerative colitis

**DOI:** 10.3389/fimmu.2025.1569271

**Published:** 2025-06-24

**Authors:** Xiaojie Fang, Jiahao Yang, Liu Yang, Yiyou Lin, Yanyan Li, Xin Yin, Xiaobing Dou, Chenyun Miao

**Affiliations:** ^1^ Department of Anorectal Surgery, Hangzhou TCM Hospital Affiliated to Zhejiang Chinese Medical University, Hangzhou, China; ^2^ Department of Pathology, Hangzhou TCM Hospital Affiliated to Zhejiang Chinese Medical University, Hangzhou, China; ^3^ School of Life Science, Zhejiang Chinese Medical University, Hangzhou, China

**Keywords:** inflammatory bowel disease, sex-specific differences, ulcerative colitis, RPS4Y1 gene, transcriptomics, metabolomics, macrophages

## Abstract

**Background:**

Ulcerative colitis (UC) has a complex etiology, and whether there are sex-related differences in its molecular mechanisms remains unclear. This study employed multi-omics analysis to explore sex-based differences in UC, aiming to provide support for personalized treatment.

**Methods:**

The GSE36807 and GSE206171 datasets from the GEO database were grouped by sex. Data were preprocessed using the R software, and DEGs identified using the limma package and key modules of WGCNA. LASSO regression was conducted to screen hub genes, ROC curves were used to evaluate diagnostic value, CIBERSORT was used to analyze immune cell proportions, and Spearman’s correlation was performed to explore associations. The single-cell dataset GSE214695 was processed using Seurat to analyze immune cell proportion differences. Histological, immunohistochemical, and metabolomic analyses were performed on the colon tissues of DSS-induced colitis model mice.

**Results:**

Thirty-seven DEGs and 47 co-expression modules were identified. LASSO regression highlighted RPS4Y1 as the core gene, which was significantly upregulated in males. Females showed higher proportions of resting NK cells and M0 macrophages but a lower number of eosinophils. RPS4Y1 expression was positively correlated with resting memory CD4+ T cells and eosinophils and negatively with M0 macrophages and resting mast cells. Macrophage function exhibited sex-based disparities. Increased immune cell infiltration was observed in female colon tissues compared with that in male colon tissues. Metabolomic analysis identified 140 sex-dimorphic metabolites, with significant alterations in glutathione metabolism.

**Conclusion:**

RPS4Y1 exhibits sex-specific expression in UC and plays a key role in immunomodulation. Mitochondrial energy metabolism contributes to sex-based macrophage differences, highlighting the importance of considering sex-specific mechanisms in UC diagnosis and individualized treatment.

## 1 Introduction

Inflammatory bowel disease (IBD), including Crohn’s disease (CD) and ulcerative colitis (UC), has emerged as a global health crisis, with UC demonstrating particularly concerning epidemiological trajectories (1). Contemporary surveillance revealed a striking incidence of 12.6/100,000 annually in the UK (2) and a Scottish prevalence exceeding 400/100,000 (3), reflecting patterns of accelerating disease burden observed worldwide. The characteristic bimodal age distribution (peaking in early adulthood and late middle age) underscores the complex pathophysiological interplay between environmental triggers, immune dysregulation, and gut microbial ecology; a triad that is further modulated by genetic susceptibility (4, 5).

Sex-specific disparities in the pathogenesis, progression, and therapeutic response of UC have garnered increasing attention in scientific research. However, much of the existing research has predominantly focused on the implications of pregnancy and childbirth (6, 7). Various sex-related differences, including both physiological and psychological dimensions, may have a similar influence on the disease (8). These variations affect multiple aspects of UC, including disease presentation, progression, complications, response to medical and surgical treatments, adherence to therapy, psychosocial functioning, and the prevalence of psychiatric comorbidities (9). In the era of personalized medicine, it is increasingly apparent that a uniform treatment regimen and medication strategy may be insufficient for some patients. Although investigating sex-specific differences in the pathogenesis of UC is crucial, the mechanisms explaining sex-related differences in UC remain undefined.

**Figure 1:**
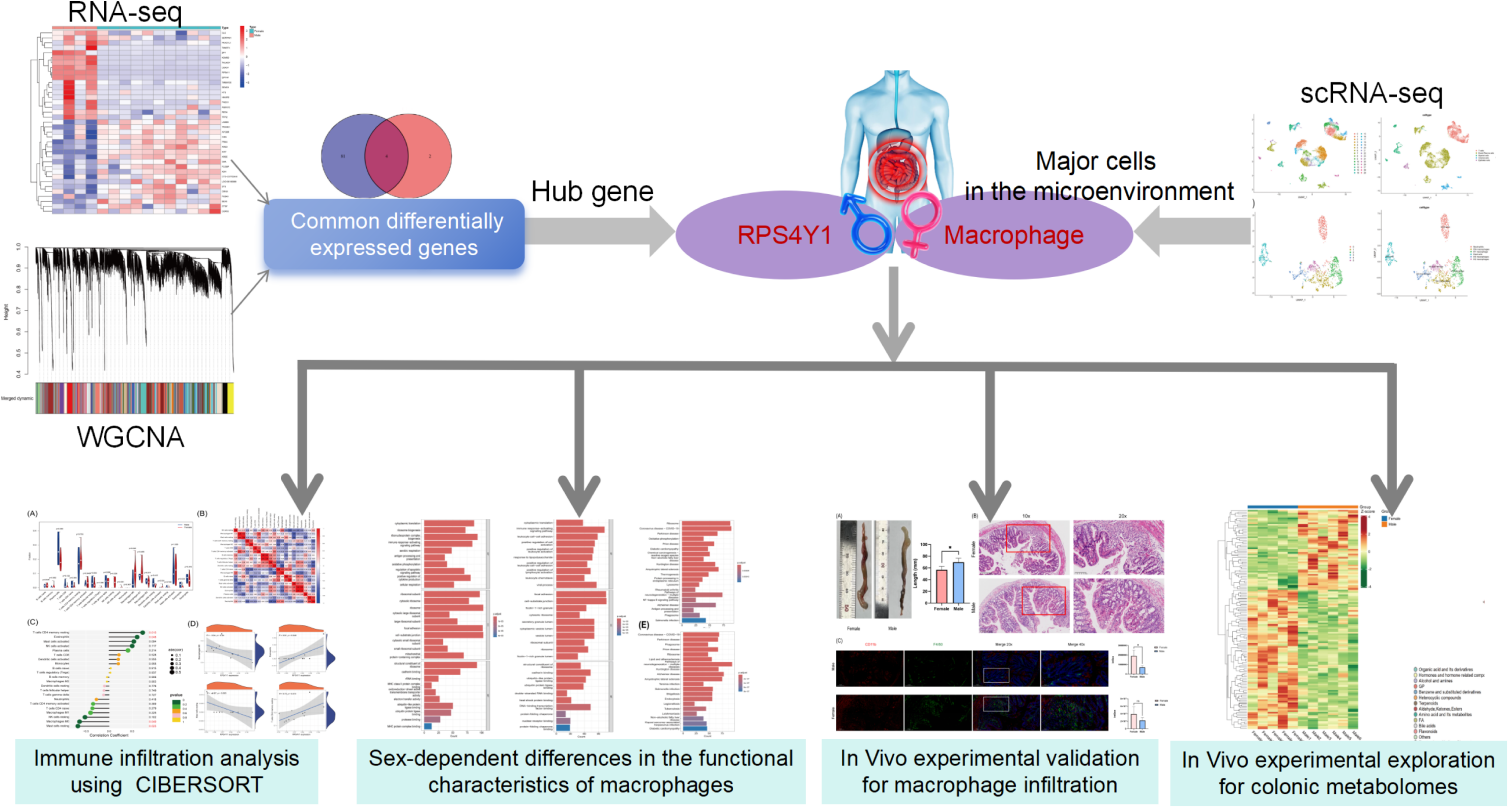
The study flowchart.

This study aimed to examine sex-related differences in the pathogenesis of UC at the transcriptomic and metabolomic levels, offering potential strategies for its precise treatment (**Figure 1**).

## 2 Materials and methods

### 2.1 Microarray dataset

An exhaustive search of the Gene Expression Omnibus (GEO) database, a widely accessible public repository established in 2020, was performed to identify relevant gene expression datasets (10). Among the identified datasets, GSE36807 and GSE206171 were selected as the most suitable for this study. The GSE36807 dataset, designated as the “training set,” consists of 7 control samples and 28 specimens from patients with IBD (**Supplementary Table S1**). To focus on disease-specific data, we excluded control data and patients with CD. Subsequently, we stratified the UC samples into male and female groups. Finally, we used the GSE206171 dataset as a validation set to verify the expression profiles of hub genes.

### 2.2 Identification and analysis of differentially expressed genes

Gene expression data were analyzed using the R Studio environment. First, data correction and normalization were performed. Subsequently, the limma package’s moderated t-test, based on the empirical parametric Bayes method, was employed to identify DEGs between male and female samples. DEGs were defined as genes with a P value < 0.05 and |Log2 (fold change (FC)) | > 1. Heatmaps were generated using the heatmap package, and volcano plots were constructed using the ggplot2 package to effectively visualize the results.

### 2.3 Weighted gene co-expression network analysis

Key modules were selected based on the correlation between the module members and gene significance. The WGCNA package in R was employed to identify hub genes. WGCNA is a robust technique used to detect clusters of highly correlated genes, referred to as modules. These modules can be effectively summarized using the module eigengene or an intramodular hub gene. WGCNA also facilitates the exploration of relationships between distinct modules and external sample traits through eigengene network analysis. Additionally, WGCNA computes module membership metrics, enabling the application of network-based gene screening methods that can assist in identifying potential biomarkers or therapeutic targets (11). Gene correlations were initially calculated, followed by the construction of a topological overlap matrix (TOM). The dissimilarity TOM (dissTOM) was calculated using the formula: dissTOM = 1 − TOM. Subsequently, a phylogenetic clustering tree was constructed via hierarchical clustering of the dissTOM to group genes with similar expression patterns into the same modules. Then, genes from the key modules were selected for further analysis.

### 2.4 Hub genes

Genes within the key modules identified via the WGCNA were subjected to comparative analysis with the DEGs (filtered by adj.P < 0.05). Genes present at the intersection of these two groups were designated as candidate hub genes. Colored Venn diagrams were then constructed using the “VennDiagram” R package. The least absolute shrinkage and selection operator (LASSO) regression was applied to the candidate hub genes to further identify those associated with UC. To ensure reproducibility, a random seed was initially set, and the “glmnet” package was utilized to handle datasets containing a large number of variables. Candidate DEGs were preprocessed and subjected to LASSO regression using the ‘glmnet’ function. The data were treated as a binary classification problem with the response variable derived from the sample names using regular expressions. Model evaluation was performed by plotting the model object and employing cross-validation with ‘cv.glmnet’ to determine the optimal lambda value. Ultimately, genes with non-zero coefficients obtained using the optimal lambda value were considered key genes linked to disease status and were outputted. This approach not only accurately selected key genes but also improved prediction accuracy by reducing model overfitting, thereby effectively supporting biomarker discovery and research into disease mechanisms.

### 2.5 Hub gene verification

The receiver operating characteristic (ROC) curve was used to evaluate the diagnostic role of hub genes in the male and female samples. First, ROC curves were plotted using the ROC package, and the area under the curve was determined. An area under curve (AUC) value > 0.6 indicated that the data was a good fit for the gene.

### 2.6 Evaluation of immune cell infiltration

The CIBERSORT algorithm, which utilizes linear support vector regression (SVR), is a widely used and reliable machine learning method for deconvoluting the expression matrix of 22 human immune cell subtypes (12). In this study, we employed the CIBERSORT algorithm to determine the relative proportions of different immune cells in the female and male UC samples. To ensure accurate results, we performed 1,000 calculations. Then, correlation analysis between the hub genes and immune cells was performed using Spearman’s correlation coefficient.

### 2.7 Single-cell RNA sequencing dataset

Single-cell RNA sequencing (scRNA-seq) data were obtained from the National Center for Biotechnology Information (NCBI) GEO database (accession number GSE214695). Public, de-identified data available via open access were not subject to local institutional review board requirements or patient consent, as permitted under the Common Rule. Tissue samples from the dataset were derived from colonic tissues. Patients with UC from the dataset were categorized into male (n = 3) and female (n = 3) groups for separate analysis. scRNA-seq was performed on the 10× Genomics platform, followed by sequencing on the Illumina NextSeq 500 (Homo sapiens) platform. Sequencing reads were assembled and aligned to the GRCh38 human reference genome using Cell Ranger version 3.1.0 (10× Genomics).

### 2.8 Data processing analysis and clustering with cell−type annotation

Expression count matrices were analyzed using the Seurat version 4.4.0 R package. Only cells with more than 200 features, fewer than 10,000 features, and no more than 20% of the total mitochondrial features were retained, following Seurat’s standard analysis principles. Normalization was performed using the log-normalization method. The top 4,000 highly variable genes (HVGs) were identified using the Seurat ‘FindVariableGenes’ function, with “vst” as the method. For cross-condition data integration and batch correction, the Seurat functions ‘FindIntegrationAnchors’ and ‘IntegrateData’ were applied. To mitigate the effects of cell cycle heterogeneity on cell clustering, each sequenced cell was given a cell cycle phase score based on expression of canonical markers using the Seurat function ‘CellCycleScoring’function, with the Seurat object as the input in addition to G1/S- and G2/M-phase-specific genes. Data were scaled according to mitochondria percentage using the ‘ScaleData’ function. Louvain clustering was performed on all cells using the ‘FindClusters’ function with the first 30 principal components (PCs) and a resolution of 1. A two-dimensional (2D) visualization of the clusters was generated using UMAP with the ‘RunUMAP’ function. Finally, the DEGs in each cluster were identified using the ‘FindAllMarkers’ function. Cell clusters in the resulting UMAP 2D representation were annotated to known biological cell types using canonical marker genes described in existing literature (13) (**Supplementary Table S2**). Differential gene expression analysis of each immune cell type between female and male groups was performed using the Wilcoxon test with the ‘FindMarkers’ function. The resulting DEGs were further analyzed using Gene Ontology (GO) and Kyoto Encyclopedia of Genes and Genomes (KEGG) pathway analyses with the ClusterProfiler 4.12.6 package.

### 2.9 Dextran sulfate sodium-induced colitis

The mice were divided into two groups based on sex: male and female groups. The mice were given drinking water containing 3% (w/v) dextran sulfate sodium (DSS) for 7 days. Afterward, the mice were euthanized by gradually increasing the concentration of carbon dioxide, followed by cervical dislocation. Then, the colons of mice were collected for use in various analyses. The severity of colitis was evaluated by monitoring changes in body weight, disease activity, colon length, and histology. The sample size for each group in all studies was five mice.

### 2.10 Daily observation and sample collection

The feeding and drinking behaviors of each mouse were documented, and daily examinations of their appearance (including vitality and hair condition) were also performed. Body weight, stool consistency, and fecal bleeding scores were recorded, and the disease activity index (14) was calculated (**Supplementary Table S3**). On the 7^th^ day, the mice were euthanized using the cervical dislocation method; the colon was promptly removed, and its length was measured. Part of the colon samples was fixed in 4% paraformaldehyde for histological examination.

### 2.11 Histology and immunohistochemistry

Colon tissue cross-sections were stained with hematoxylin and eosin (HE) to observe changes in colon structure. For immunofluorescence staining of paraffin-embedded colon tissues, the tissues were sectioned, dewaxed, subjected to antigen retrieval, and blocked with an immunostaining blocking solution. To assess macrophage infiltration, slides were incubated overnight at 4°C with anti-F4/80 (1:200 dilution; #28463-1-AP; Proteintech, China) and anti-CD11b (1:200 dilution; #YT5923; Immunoway, China) along with their respective secondary antibodies. Finally, the sections were stained with 4’,6-diamidino-2-phenylindole (DAPI) for nuclear staining and then observed and photographed using an Olympus microscope. All immunofluorescence images were captured using the same exposure and intensity settings.

### 2.12 Non-targeted metabolomic analysis

Non-targeted metabolomic analyses were performed by Maiwei BioTech Co., Ltd. (Wuhan, China). In brief, 400 uL of solution (MeOH: H_2_O= 7: 3, V/V) was added to 20 mg of the sample, shaken (1,500 rpm and 5 min), iced (15 min), and centrifuged (12,000 rpm, 10 min, and 4°C). Subsequently, 300 μL of supernatant was collected and stored at -20°C for 30 min and centrifuged again (12,000 rpm, 3 min, 4°C). Metabolites were quantified using a liquid chromatography-electrospray ionization-tandem mass spectrometry (LC-ESI-MS/MS) system (15). For data analysis, unsupervised PCA was conducted using ‘prcomp’ in R, after unit variance scaling. HCA and PCC were performed using the ComplexHeatmap R package, presenting HCA as heatmaps with dendrograms and PCC as heatmaps alone. For 2-group analysis, differential metabolites were identified using VIP (VIP > 1) and P < 0.05 (t-test). VIP values were obtained from the OPLS-DA results (MetaboAnalystR R package) after log transformation and mean centering, followed by a 200-permutation test. Identified metabolites were annotated using the KEGG Compound DB and mapped to the KEGG Pathway DB.3.

## 3 Results

### 3.1 Identification of DEGs between the male and female groups

**Figure 2:**
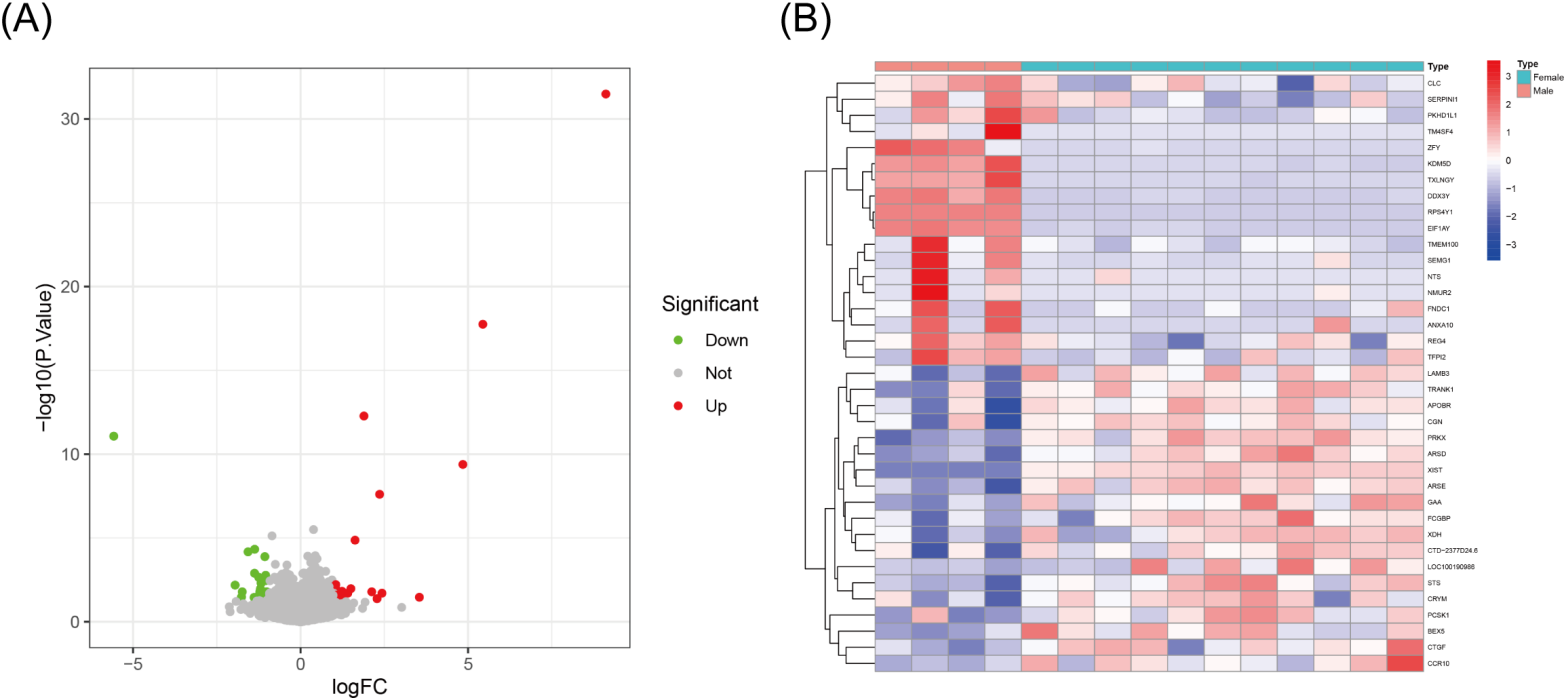
Sex-specific differential gene expression in ulcerative colitis (UC). **(A)** Volcanic map of differential gene expression in UC. **(B)** Heat map analysis of differential gene expression in UC.

We identified a total of 21,654 DEGs between the male and female samples. Compared to the female samples, we applied the conditions to the data and ultimately obtained 37 DEGs from the male samples. Among these, 19 genes were downregulated and 18 genes were upregulated in the male samples (**Supplementary Table S6**). A heatmap and volcano plot of the DEGs are shown in **Figure 2**.

### 3.2 WGCNA and co-expressed genes

**Figure 3:**
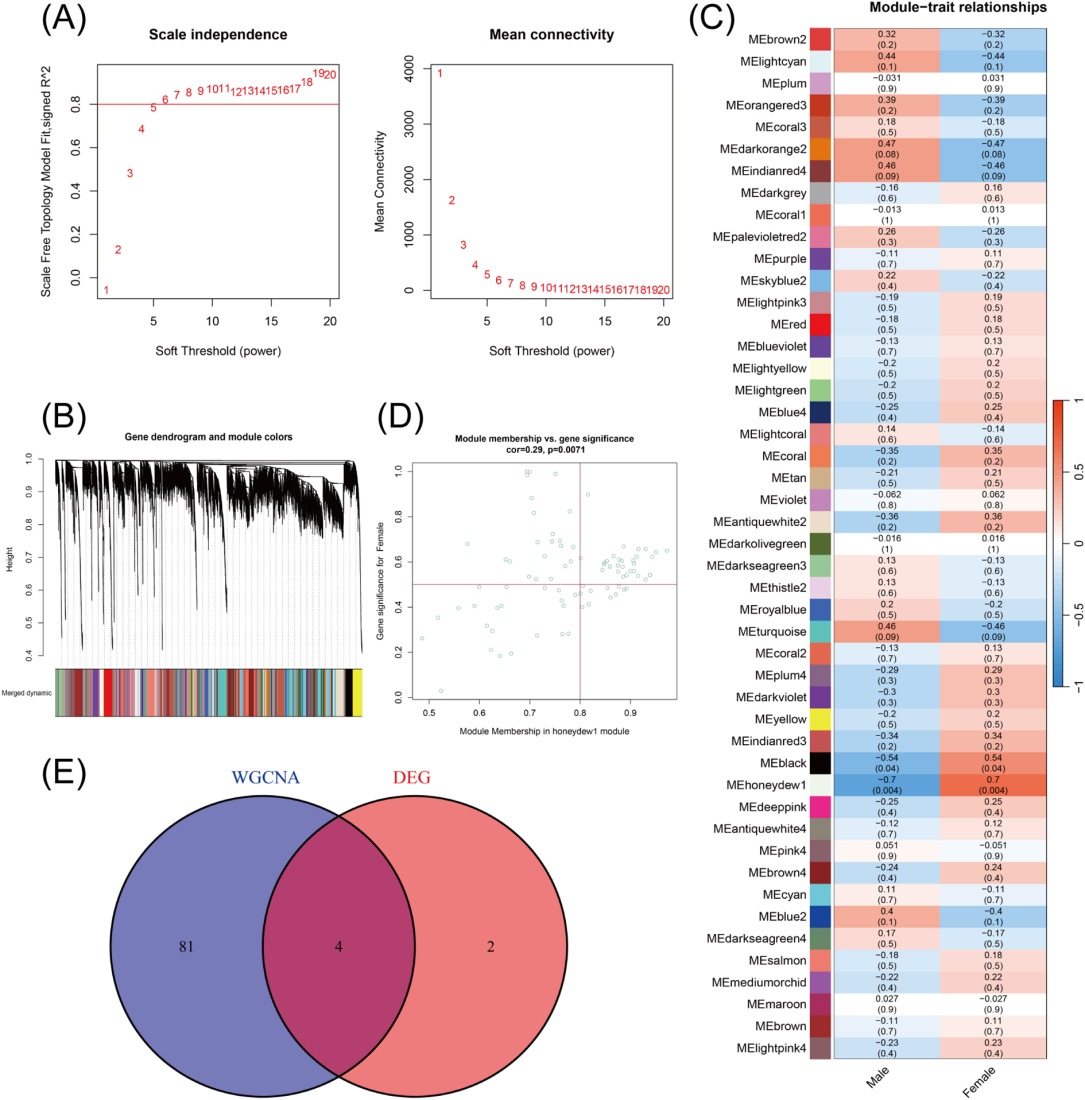
The WGCNA analysis and identification of candidate hub genes. **(A)** The soft threshold power and the mean connectivity of WGCNA. **(B)** The cluster dendrogram of WGCNA. **(C)** The clustered modules of WGCNA. **(D)** Correlation analysis between key modules and gene importance in WGCNA. **(E)** The venn diagram showed the interactions between the DEGs and genes in the MEhoneydew1 module.

A sample clustering tree was constructed, and a soft threshold of β = 8 was set (**Figure 3A**). The dynamic tree cut method was employed to initially identify the modules, and similar modules were merged. The minimum number of genes per network module was set to 20, and ultimately, 47 modules were identified. The grey modules could not be aggregated with the other modules (**Figure 3B**). Based on the module-trait associations (**Figure 3C**), the MEhoneydew1 module was selected as a key module (R = 0.7, P = 0.004). 85 genes were identified within the honeydew1 module (**Figure 3D**) (**Supplementary Table S6**). Intersecting the DEGs with the genes from the MEhoneydew1 module identified through WGCNA revealed 4 co-expressed genes (**Figure 3E**).

### 3.3 Verification of hub genes

**Figure 4:**
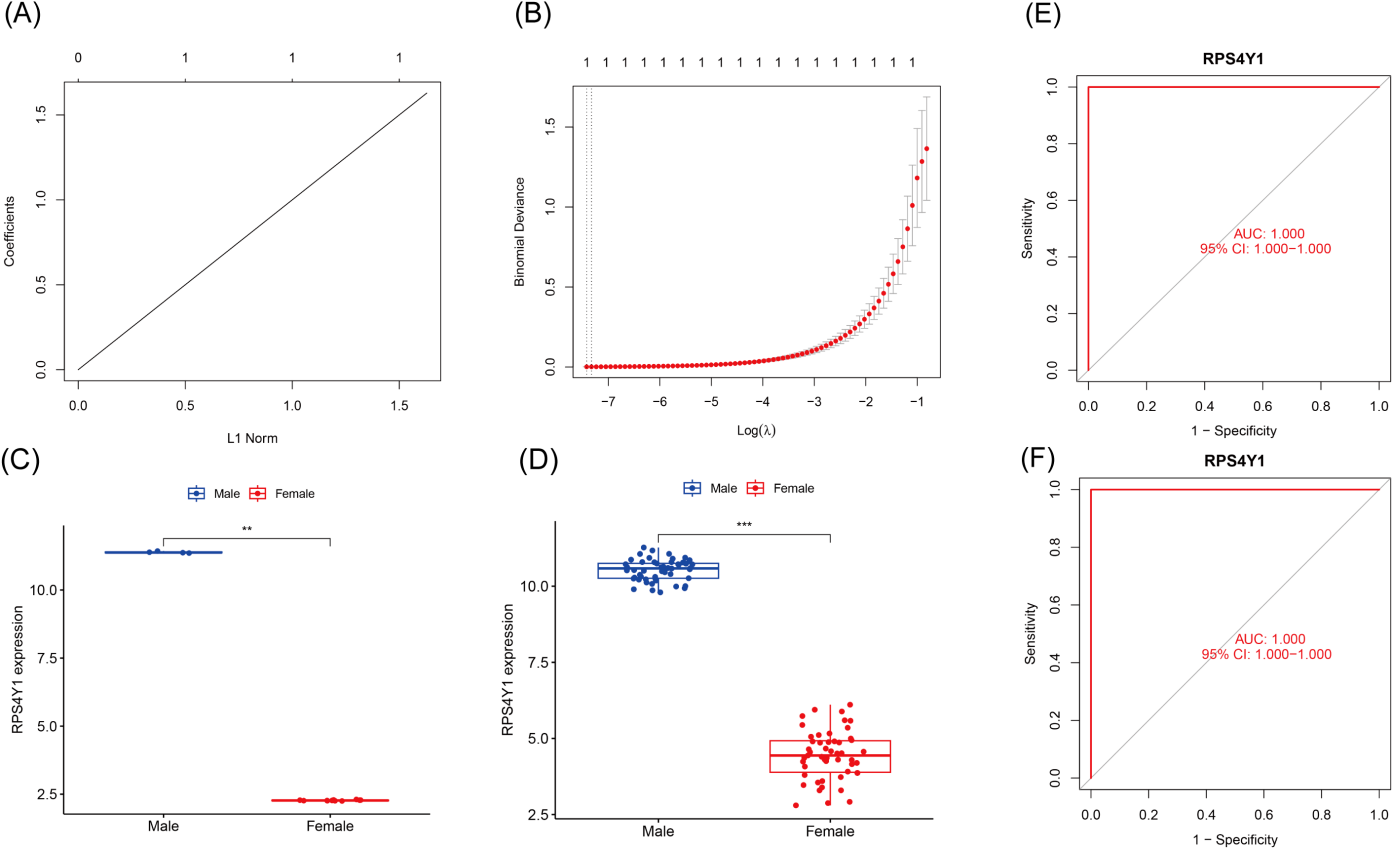
Biomarkers screened by the Least Absolute Shrinkage and Selection Operator (LASSO) regression algorithm. **(A)** LASSO coefficient profiles of 4 andidate hub genes. **(B)** Selection of the optimal lambda value in the LASSO model. **(C)** The expression levels of the RPS4Y1 in training set. **P < 0.01. **(D)** The expression levels of the RPS4Y1 in validation set. ***P < 0.001. **(E)** ROC curve of RPS4Y1 in training set. **(F)** ROC curve of RPS4Y1 in validation set.

To identify hub genes, LASSO regression analysis was performed on the genes shared between the WGCNA and DEGs (**Figures 4A, B**). Through the LASSO analysis, ribosomal protein S4 Y-linked 1 (RPS4Y1) was identified as the key hub gene. The accuracy of the identified hub gene was validated by investigating its expression levels in both the training and validation sets. RPS4Y1 expression was observed to be significantly upregulated in males. RPS4Y1 likely plays a crucial role in the sex-related disparities among patients with UC (**Figures 4C, D**). Furthermore, the AUC was calculated for RPS4Y1. The objective of this study was to assess the sex-discriminating diagnostic accuracy of RPS4Y1 in patients with UC and determine whether it could serve as an effective biomarker. Notably, the AUC values for RPS4Y1 were consistently 1.0, indicating its potential to distinguish between sexes (**Figures 4E, F**).

### 3.4 Analysis of immune infiltration

**Figure 5:**
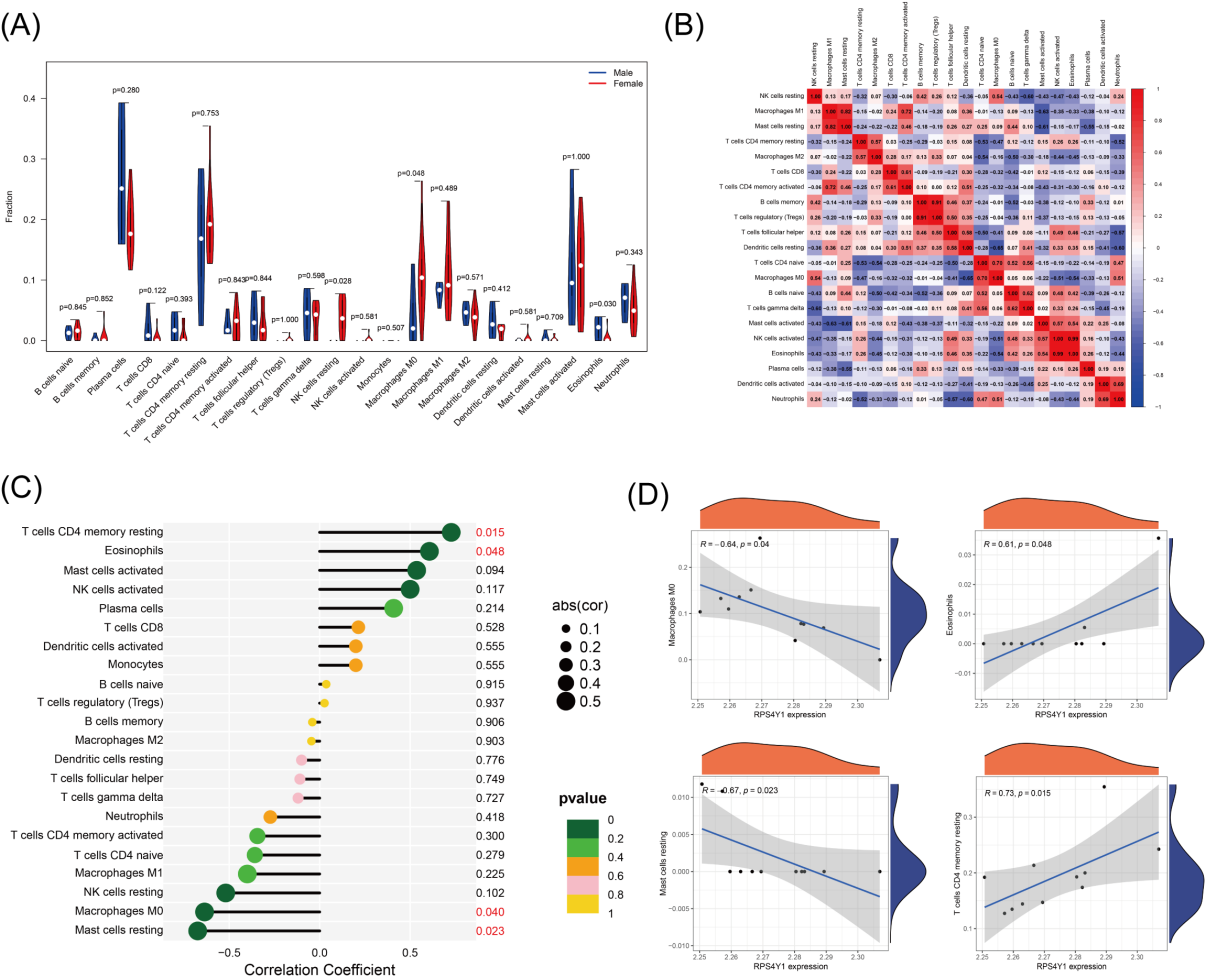
The immune related analysis in ulcerative colitis (UC). **(A)** The difference of 22 immune infiltration between Male and Female groups, quantified using CIBERSORT deconvolution algorithm. **(B)** The correlation among different immune cell types. **(C)** Lollipop map of the correlation between RPS4Y1 and immune cells (Values with P < 0.05 have been marked in red). **(D)** RPS4Y1 expression was negatively associated with Mast cells resting and Macrophages M0, but positively associated with infiltration of T cells CD4 memory resting and Eosinophils.

The analysis of immune infiltration revealed substantial differences in immune cell infiltration between sexes in patients with UC. Specifically, female patients exhibited higher infiltration of resting NK cells (P = 0.028) and M0 macrophages (P = 0.048) and lower infiltration of eosinophils (p = 0.030) compared with male patients (**Figure 5A**). These findings suggest that resting NK cells, M0 macrophages, and eosinophils may significantly contribute to sex disparities in UC. Analysis of immune cell infiltration revealed notable associations between the hub gene (RPS4Y1) and specific immune cell types in male and female groups. RPS4Y1 exhibited positive correlations with resting memory CD4+ T cells and eosinophils and negative correlations with M0 macrophages and resting mast cells (**Figures 5C, D**).

### 3.5 Single-cell sequencing revealed the major cell types in the microenvironment of UC

**Figure 6:**
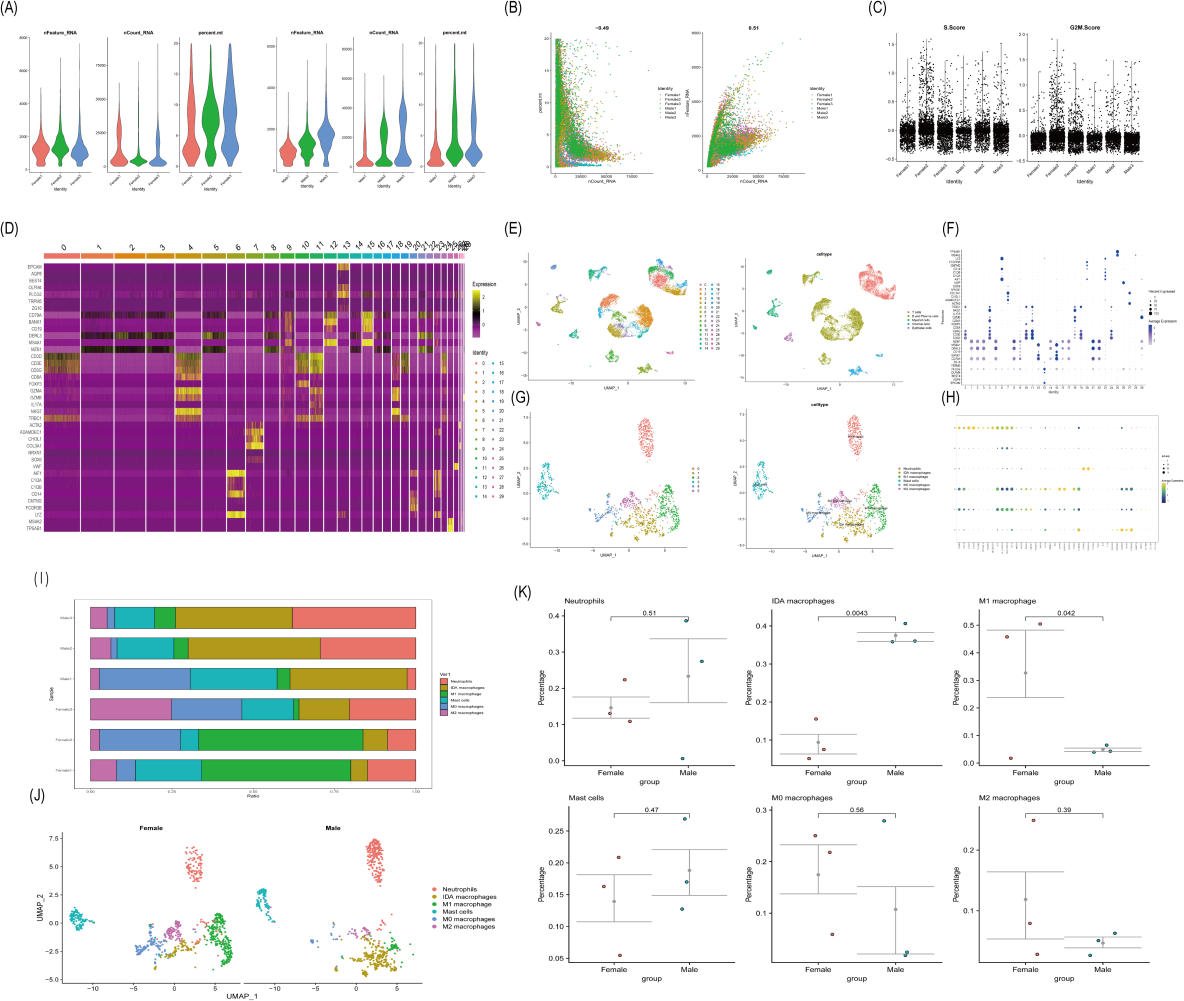
Proportion mapping of UC-associated cell populations by scRNA-seq. **(A)** After quality control, the scatter plot shows the number of genes, the percentage of UMI, and mitochondrial genes in each cell type from Female and Male groups. **(B)** Relationships between mitochondrial gene percentage and mRNA reads and between mRNA quantity and reads. **(C)** Cell cycle stage scores for samples from the Female and Male groups. **(D)** The heat map of the gene clustering of cell subpopulations. **(E)** Cell clustering and annotation. **(F)** Marker gene expression in each cluster. **(G)**UMAP visualization of myeloid cell subclustering **(H)** Expression of marker genes in each myeloid cell subpopulation. **(I)** Stacked bar plots showing the percentage of major myeloid cell types in different samples. **(J)** UMAP plot visualization of the myeloid cell types between Female and Male. **(K)** Differences in the proportion of myeloid cells in the Female and Male groups.

To identify the major cell types in the UC tissue microenvironment, we obtained single-cell sequencing data from the GEO database (GSE214695) for 6 patients with UC (3 male and 3 female patients). After rigorous quality control and batch correction, we downscaled the data using linear and nonlinear approaches and clustered the cells based on intercellular distances without bias. A total of 15,280 cells were grouped into 30 major clusters (**Figures 6A-D**). To identify the cell type of each cell cluster, we calculated the characteristic genes for each cluster based on the clustering results of the cell clusters (**Supplementary Table S7**). We compared the characteristic genes with relevant literature and annotated the cells in UC tissues, identifying five major cell types: T cells (CD3D, CD3E, CD3G), B and plasma cells (CD79A, DERL3, MZB1), myeloid cells (AIF1, CD14, LYZ, MS4A2, TPSAB1), stromal cells (ACTA2, COL3A1, VWF) and epithelial cells (EPCAM, OLFM4) (**Figures 5E, F**). Using CIBERSORT immune cell infiltration results, we further subdivided the myeloid cells. A total of 7,885 myeloid cells were clustered into 6 subpopulations: neutrophils (PROK2, CXCL8, FCRG3B, AQP9, S100A8, S100A9), inflammation-dependent alternative (IDA) macrophages (NRG1, AREG, EREG, HBEGF), M1 macrophages (ACOD1, TNIP3, IL1B, INHBA, IL6, VCAN, CXCL5, CD300E), mast cells (LTC4S and TPSB2), M0 macrophages (HLA-DPB1, HLA-DPA1, SELENOP), and M2 macrophages (CD209, CD163L1, FOLR2) (**Figures 6G, H**).

### 3.6 Assessment of intragroup differences in cell composition

The proportion of each cell type in the two sample groups is shown in **Figure 6I**. The relative abundances of the six major myeloid cell populations varied between the groups (**Figure 6J**). Generally, compared with that of the male group, the percentage of IDA macrophages in the female group decreased (P = 0.0043), while that of M1 macrophages increased (P = 0.042), with no significant differences observed in the remaining four cell types. These results indicate significant differences in macrophage immune infiltration in UC tissues between different sexes (**Figure 6K**).

### 3.7 Sex-dependent differences in the functional characteristics of macrophages within UC colon tissues

**Figure 7:**
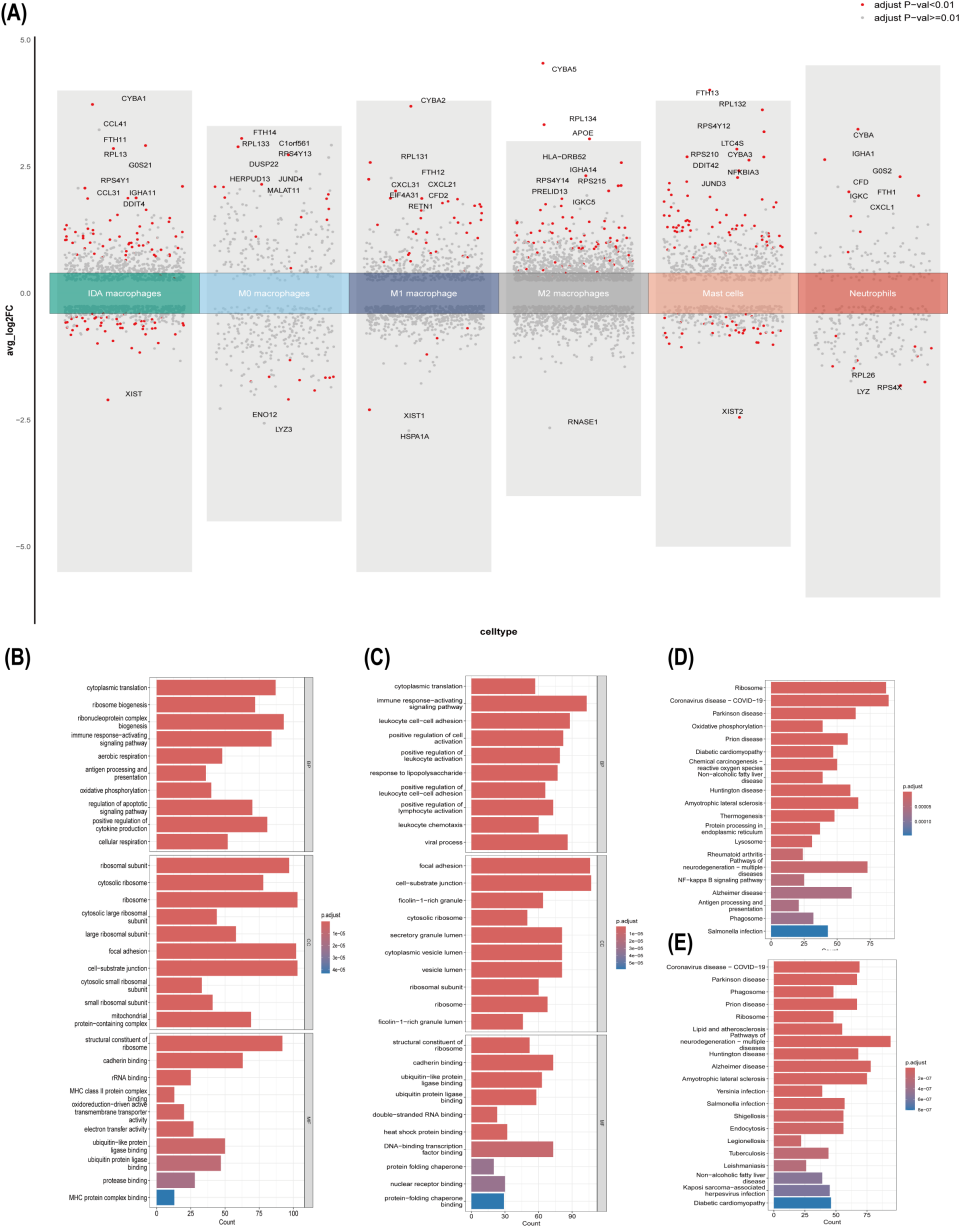
Functional enrichment analysis at the level of single-cell subpopulations. **(A)** Single-cell heatmap of differentially expressed genes, with genes above the x-axis being highly expressed and those below being lowly expressed. **(B)** GO enrichment analysis of sex-specific differential genes of IDA macrophages. **(C)** GO enrichment analysis of sex-specific differential genes of M1 macrophages. **(D)** KEGG enrichment analysis of sex-specific differential genes of IDA macrophages. **(E)** KEGG enrichment analysis of sex-specific differential genes of M1 macrophages.

To further explore the mechanisms underlying the differences in myeloid cell infiltration, we conducted differential gene analysis on myeloid cells in each group. As shown in **Figure 7A**, the core differential gene RPS4Y1 (P < 0.01) was significantly differentially expressed in IDA macrophages.

Based on these results, we performed GO term and KEGG pathway enrichment analyses on the significant differential genes in IDA and M1 macrophages from both groups (**Supplementary Table S7**). The GO results revealed that differences in IDA macrophages were primarily associated with immune response-activating signaling pathways, positive regulation of cytokine production, antigen processing and presentation, cellular respiration, aerobic respiration, oxidative phosphorylation, and oxidoreduction-driven active transmembrane transporter activity. In contrast, differences in M1 macrophages were mainly associated with the immune response-activating signaling pathway, leukocyte cell-cell adhesion, positive regulation of leukocyte activation, positive regulation of lymphocyte activation, positive regulation of leukocyte cell-cell adhesion, leukocyte chemotaxis, cadherin binding, ubiquitin-like protein ligase binding, ubiquitin protein ligase binding, and heat shock protein binding. The KEGG results showed that the differences in IDA macrophages were primarily related to oxidative phosphorylation, NF−κB signaling, and antigen processing and presentation, while differences in M1 macrophages were mainly related to Yersinia infection, phagosome, salmonella infection, shigellosis, endocytosis, legionellosis, and tuberculosis (**Figure 7B**).

### 3.8 Macrophage infiltration within UC colonic tissues exhibits sex-dependent variation

**Figure 8:**
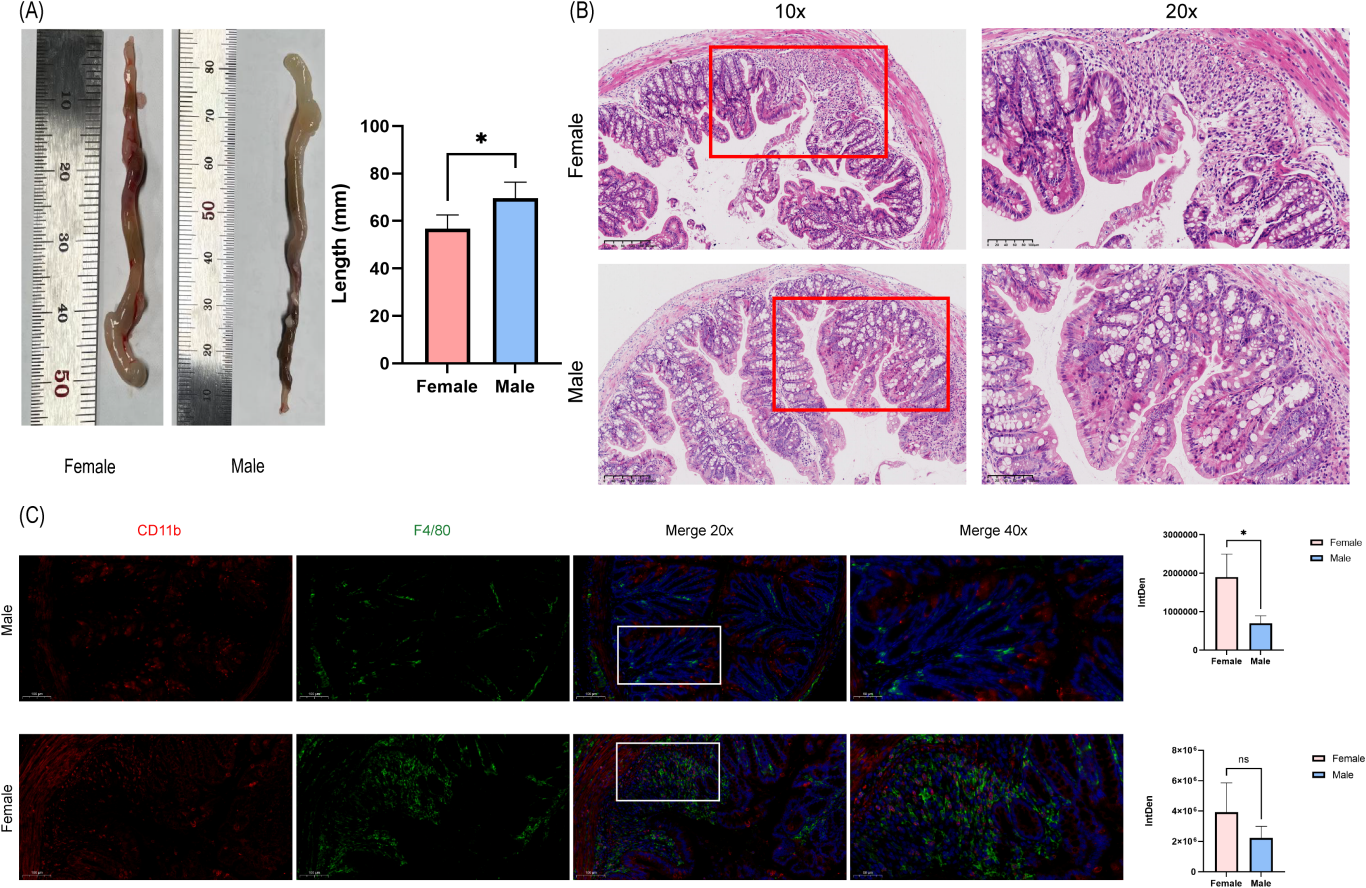
HE and immunofluorescence staining of DSS-induced colitis mice colon tissues. **(A)** Representative colon length photographs and quantitative comparison between two groups. *P<0.05. **(B)** HE staining of colon samples from DSS mice and comparison of tissue inflammatory cell infiltration between the two groups. **(C)** Immunofluorescence dual-staining showing spatial distribution of F4/80+ macrophages (green) and CD11b+ monocytes (red) in lamina propria, with quantitative analysis of fluorescence integrated density (ID). Cell nuclei are counterstained with DAPI (blue). Asterisks denote statistically significant differences (*P<0.05) determined by two-tailed t-test between Male and Female groups (n=4–5 animals per group). ns, not significant.

To validate the results of immune cell infiltration and compare the degree of tissue inflammation, we conducted HE staining of colonic tissues. Macroscopically, female mice exhibited significantly reduced colon lengths compared with those of males (P < 0.05) (**Figure 8A**). The results of HE staining showed that, compared to the male group, the female group exhibited a greater infiltration of lymphocytes and eosinophils in the mucosal, muscular, and serosal layers of the colonic tissues. In the male group, only some inflammatory cell types infiltrated the tissues, and these were mostly confined to the mucosal layer (**Figure 8B**). Furthermore, double immunofluorescence staining of CD11b and F4/80 expression, we confirmed a higher degree of macrophage infiltration in the female group compared with that in the male group (P<0.05) (**Figure 8C**).

### 3.9 Sex-related differences in mice colonic metabolomes

**Figure 9:**
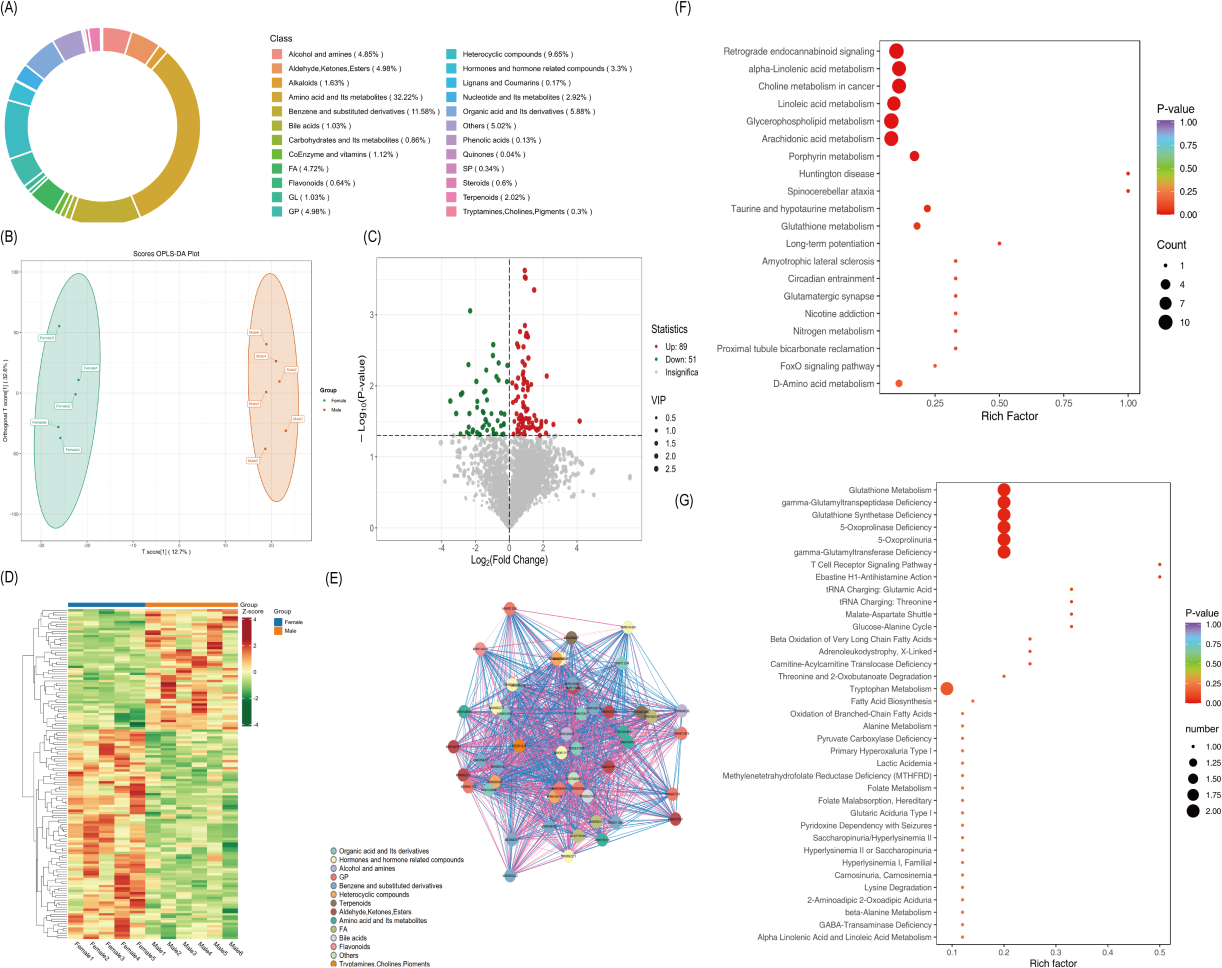
Untargeted metabolomics of DSS-induced colitis mice colon tissues. **(A)** Relative abundance distribution of major metabolite identified in colonic tissues. **(B)** The score plots from OPLS-DA showed significant metabolomic differences between Female and Male groups. (R^2^X=0.552, R^2^Y=0.994, Q^2^ = 0.367; permutation test with 200 iterations). **(C)** Volcano plot identifying 140 differential metabolites (red: upregulated, green: downregulated), differential metabolites were determined by VIP (VIP > 1) and P value (P < 0.05, Student’s t test). **(D)** Heat map of differential metabolite clustering (red for high levels, green for low levels). **(E)** Differential metabolite correlation network diagram (red lines represent positive correlations, blue lines represent negative correlations. The thicker the line, the greater the Pearson correlation coefficient). **(F)** Kyoto Encyclopedia of Genes and Genomes (KEGG) pathway enrichment with bubble size indicating number of mapped metabolites and color intensity representing -log10(P-value). **(G)** HMDB-based metabolic superclass enrichment analysis with bubble size corresponding to pathway impact value and color scale reflecting enrichment factor.

Next, we employed a non-targeted metabolomics technique to characterize the colonic tissue metabolome of mice. A total of 5,043 metabolites were identified in colonic tissue samples from both female and male mice (**Figure 9A**). Then, we constructed OPLS-DA models to compare metabolite patterns between the female and male colonic tissues (**Figure 9B**). A total of 140 metabolites exhibited significantly different levels [variable importance in the projection (VIP, which computes the influence on Y of each term in the model summed over all model dimensions) >1, adjusted P < 0.05], as identified using the OPLS-DA model (**Supplementary Table S4**, **Figure 9C**). The differentially regulated metabolites primarily consisted of amino acids, benzene and its substituted derivatives, aldehydes, ketones, esters, fatty acyl groups, and their intermediates (**Figures 9D, E**). These altered metabolites were further annotated using the Human Metabolome Database (HMDB) and KEGG analysis, which identified 140 differentially regulated metabolites across the female and male mice, which could be matched to all three databases. Pathway enrichment analysis of these metabolites using Metabolites Biological Role (MBROLE) and MetaboAnalyst revealed significant changes in metabolites associated with redox signaling-related metabolic pathways and enzymes, particularly the glutathione metabolism pathway (**Figures 9F, G**).

## 4 Discussion

In this study, we initially employed transcriptomic data analysis to identify DEGs across sexes in patients with UC. Notably, RPS4Y1, located on the sex chromosome, emerged as a key gene that is closely associated with the immune microenvironment of UC. Subsequently, single-cell transcriptomic techniques were used to analyze disparities in immune cell composition within the sex-stratified UC samples. Furthermore, *in vivo* murine experiments were performed to validate that macrophages were the central factor underlying sex-related discrepancies in UC. Additionally, metabolomic profiling of colonic tissues was performed using UC mouse models of both sexes, revealing that mitochondrial energy metabolism likely plays a key role in macrophage-related differences.

Macrophages are widely recognized as critical factors in the progression of UC (16). Most steady-state colonic mouse macrophages are formed through the CCR2-dependent infiltration of LY6Chi blood-derived monocytes, where CCR2 stands for CC-chemokine receptor 2 (17). Defects in resolving intestinal inflammation are causally linked to altered monocyte-macrophage differentiation (16). Previous studies suggested that IBD susceptibility loci are highly enriched in promoters involved in macrophage activation and differentiation (18).

Macrophages, as phagocytic cells, can alter their phenotype in response to the body’s internal environment, particularly in response to sex hormones (19). Estrogen receptor (ER) signaling promotes the formation of anti-inflammatory macrophage phenotypes by regulating cytokine production, phagocytic activity, and chemotaxis (20). Knocking out androgen receptors (ARs) in monocytes/macrophages leads to a reduction in lung inflammation and eosinophil recruitment to inflammatory sites in male mice, but this effect is not observed in female mice (21). In IBD research, previous studies have reported findings consistent with our observations. Female mice have a higher intestinal macrophage proportion compared with that of male mice (67.9% vs. 141.2%) (22). In female rats, estrogen and progesterone reduced the production of macrophage migration inhibitory factor (MIF), which promotes tumor necrosis factor (TNF)-alpha and interleukin-1β (IL-1β) expression in early colitis (23).

M1 macrophages are primarily induced by interferon-γ (IFNγ) and secrete pro-inflammatory cytokines, such as interleukin-12 (IL-12), tumor necrosis factor-α (TNFα), IL-1β, and interleukin-6 (IL-6). They are metabolically characterized by fatty acid storage, reduced oxidative phosphorylation (OXPHOS), and increased nitric oxide (NO) production (24, 25). In contrast, M2 macrophages are activated by interleukin-4 (IL-4) and interleukin-13 (IL-13), secreting anti-inflammatory cytokines including interleukin-10 (IL-10) and transforming growth factor-β (TGFβ). They also exhibit increased OXPHOS and fatty acid β-oxidation (26, 27). Consequently, mitochondrial energy metabolism plays a critical role in macrophage polarization (28). Moreover, non-targeted metabolomics of UC model mice revealed that glutathione metabolism is pivotal in UC. Mitochondrial glutathione (mGSH) serves as a redox regulator for OXPHOS and facilitates the sequential transfer of electrons among OXPHOS complexes within the inner mitochondrial membrane. Additionally, mGSH is of crucial importance for redox signaling and the biosynthesis of iron-sulfur (Fe-S) cluster cofactors in the mitochondrial matrix. The regulation of OXPHOS proteins implies a potential link between mitochondrial metabolism and redox homeostasis, which might be mediated by the status of mGSH (29, 30).

Both the hormonal environment and the immune system are characterized by complexity and diversity, with sex steroids serving as key modulators of sex differences in immune function. The primary sex steroid hormones are estrogen, progesterone, and testosterone. These sex hormones bind to specific cell membranes and/or nuclear-related receptors located either on the surface or within immune cells, thereby influencing gene expression and cell functions (31). Specifically, in both preclinical and clinical scenarios, a bidirectional interaction may occur between sex steroids and the disease state. A previous cross-sectional study investigated the gender differences in the prevalence of 31 autoimmune diseases, over 80% diseases were higher in females than in males. Among them, the highest prevalence ratios were observed in autoimmune thyroiditis (PR 5.92; 99% confidence interval [CI]: 5.88–5.95), primary biliary cirrhosis (5.60; 5.36–5.84), and systemic lupus erythematosus (5.15; 4.97–5.36) (32). Sex hormone receptor signaling modulates mitochondrial dynamics by localizing to mitochondria and regulating the expression of mitochondrial genes, such as dynamin-related protein 1 and mitofusins (33, 34). Published *in vivo* studies have shown that estrogen is crucial for maintaining healthy cardiac function by regulating mitochondrial processes (35). Upon activation, estrogen-related receptors enhance the expression of genes related to fatty acid oxidation, respiratory chain activity, and mitochondrial dynamics (36).

RPS4Y1 encodes ribosomal protein S4, located on chromosome p11.31 (37). Although direct evidence linking RPS4Y1 to IBD is lacking, studies have shown its association with immune infiltration in idiopathic pulmonary fibrosis (38), multiple sclerosis (39), and esophageal squamous cell carcinoma (40). Various studies have indicated that RNA modification plays an essential role in innate immunity and inflammation. The inhibition of ribosomal RNA (rRNA) transcription and synthesis induces apoptosis and cell cycle arrest, both of which are key regulators of immune responses (41). Based on these findings, the RNA transcription process regulated by RPS4Y1 represents a potential direction for future research.

This study provided a comprehensive analysis of the biological mechanisms underlying sex disparities in UC by integrating multi-omics strategies, thereby revealing the influence of sex on the immune and metabolic landscapes of UC. The results of this study provide potential insights for personalized and precision treatment in the clinical management of UC. First, our findings validate the existence of gender differences in UC and identify that the RPS4Y1 gene may play a key role in the gender-specific manifestations of UC. Additionally, most currently available biologic agents such as Adalimumab and Infliximab are inhibitor for TNF-α, which is secreted by M1 macrophage. When using these drugs, it should be noted that the dosage for females should differ from that for males due to the different levels of M1 macrophage polarization between the two genders. However, this study has some limitations. Notably, the sample size of the single-cell omics data was relatively small, which may affect the reliability of the results to a certain extent because of the low sequencing accuracy and sampling error. The UC animal models induced by DSS does not fully replicate the physiological reactions observed in human UC (42). Human UC is influenced by a combination of environmental, genetic, and immunological factors (43). In contrast, DSS-induced inflammation is more severe and progresses more rapidly than the inflammation observed in human UC. Additionally, DSS colitis is characterized by indiscriminate epithelial cell damage and extensive microbial invasion into the lamina propria, features that are uncommon in human UC (44). Immunologically, chronic DSS colitis exhibits mixed T-cell pro-inflammatory cytokine responses, contrasting with the polarized T-cell responses typical of human chronic UC (45). Moreover, clinical samples were not included to validate the differential expression of macrophages, limiting the generalizability of the findings across species. Additionally, the mechanism of RPS4Y1 and macrophage energy metabolism were not verified in this study, upcoming research should aim to confirm our results in human samples.

## 5 Conclusion

RPS4Y1, a gene exhibiting sex-specific expression in patients with UC, also plays a pivotal role in immunomodulation. We identified mitochondrial energy metabolism associated with RPS4Y1 as a potential contributor to sex-based differences in macrophage function in UC. These findings elucidate the molecular mechanisms underlying sex-specific variations in UC pathogenesis and highlight a promising avenue for the development of sex-tailored therapeutic strategies.

## Data Availability

The datasets presented in this study can be found in online repositories. The names of the repository/repositories and accession number(s) can be found in the article/**Supplementary Material**.
